# Femoral nerve compression secondary to a ganglion cyst arising from a hip joint: a case report and review of the literature

**DOI:** 10.1186/1752-1947-3-33

**Published:** 2009-01-29

**Authors:** Aydıner Kalacı, Yunus Dogramaci, Teoman Toni Sevinç, Ahmet Nedim Yanat

**Affiliations:** 1 Department of Orthopaedics and Traumatology, Mustafa Kemal University Faculty of Medicine, 31100 Antakya, Hatay, Turkey

## Abstract

**Introduction:**

Femoral nerve compression due to a cystic lesion around the hip joint is rare and only a few cases have been described in the literature. Among these, true ganglion cysts are even more rare.

**Case presentation:**

We report the case of a 57-year-old woman with femoral nerve compression caused by a true ganglion cyst of the hip joint.

**Conclusion:**

A high index of suspicion is required to predict a non-palpable cystic lesion around the hip joint as it may mimic different disorders and should be kept in mind in the differential diagnosis of unusual groin pain, radicular pain and peripheral vascular disorders.

## Introduction

Peripheral nerve compression secondary to peri-articular cysts has been described in the lower limb, including tibial nerve compression secondary to popliteal cysts and common peroneal nerve compression secondary to cysts of the tibio-fibular joint. Posterior tibial nerve compression may occur within the tarsal tunnel secondary to intraneural ganglia (tarsal tunnel syndrome). Femoral nerve compression due to a cystic lesion around the hip joint is rare and only very few cases have been described in the literature [1-5]. Among these, true ganglion cysts are even rarer.

In this study, we report on a woman suffering femoral nerve compression caused by a true ganglion cyst arising from the hip joint. Surgical resection resulted in complete resolution of the symptoms in the early postoperative period.

## Case presentation

A 57-year-old woman presented with a 6 month history of increasing pain in the right side of her groin radiating down to the medial thigh, to the anterior aspect of the knee and to the medial side of the lower leg and foot. The pain worsened when climbing stairs and walking and could not be relieved by non-steroidal medications. She became unable to climb stairs and walk long distances for the preceding 2 months, due to paresthesias and hypoesthesia of the medial side of the right lower leg and foot.

Physical examination revealed tenderness over the anterior hip, no palpable mass and there was no muscle atrophy. Neurological examination revealed an altered sensation to light touch in the medical side of the right lower leg and foot. Internal rotation, external rotation and extension of the right hip were painful and were markedly limited. Vascular examination of her lower limb was intact. The result of the straight-leg-raising test was negative.

**Figure 1:**
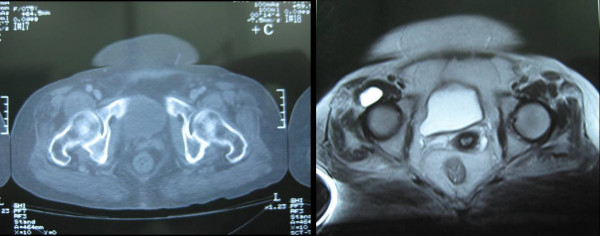
**Computer tomography scan and magnetic resonance imaging of the right hip region revealed a cystic lesion of 2.6 × 1.4 cm in diameter, displacing the femoral nerve as well as the femoral vein and artery**.

Laboratory investigation included blood sugar, serum electrolytes, erythrocyte sedimentation rate, C-reactive protein, corpuscular blood count with differential count, rheumatoid factor and antinuclear antibodies. The results were all normal. Radiographs of the hip and lumbar spine were also normal. Computer tomography (CT) scan and magnetic resonance imaging (MRI) of the right hip region revealed a cystic lesion of 2.6 × 1.4 cm in diameter, both in high-T2- and low-T1-weighted signal intensity, arising from the antero-medial aspect of the acetabulum, displacing the femoral nerve as well as the femoral vein and the artery (Figure 1). A colour duplex Doppler ultrasonography excluded the possibility of aneurismal lesion. Electromyography (EMG) revealed evidence of muscle denervation.

**Figure 2:**
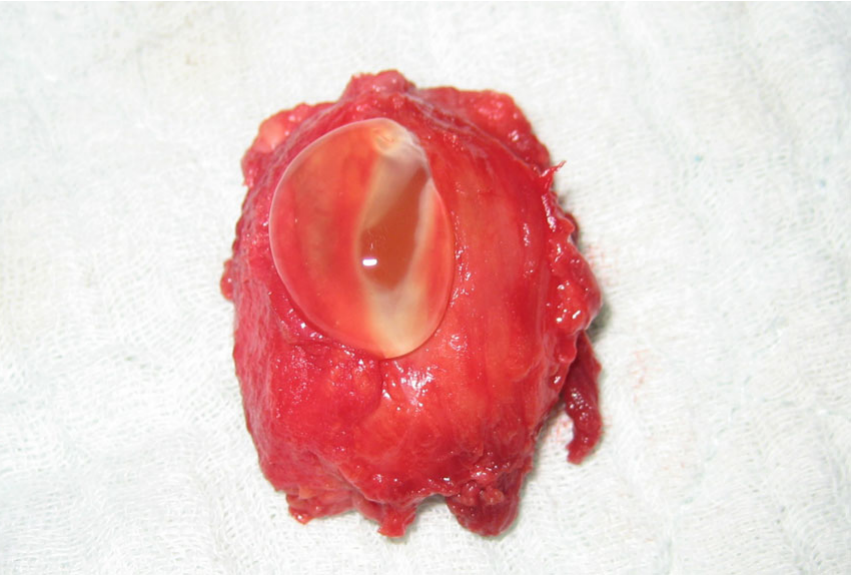
**Excision showing the cyst to contain a thick lucent gelatinous material**.

At the time of surgery, a cyst measuring 3.3 × 2.4 × 1.8 cm was identified which was adherent to the anterior surface of the hip joint capsule. No communication was found between the joint space and the cyst. The joint capsule appeared intact. The cyst was removed totally and the base of the cyst was cauterized. The cyst was opened in the operating room and was found to contain thick, lucent gelatinous material (Figure 2).

**Figure 3:**
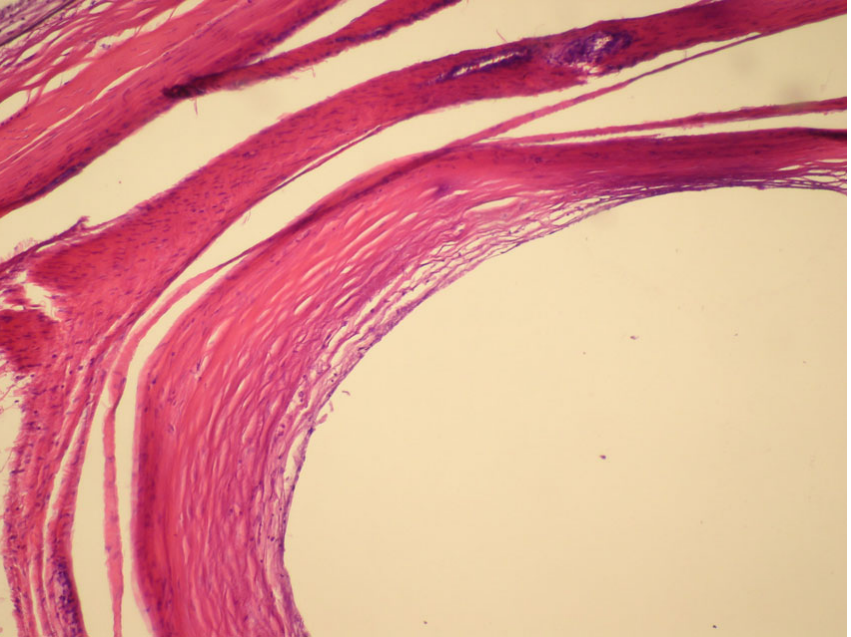
**Pathologic specimen showing benign cyst wall composed of variably dense fibroconnective tissue with no lining cells, consistent with a ganglion cyst**.

Histological evaluation identified fragments of benign cyst wall composed of variably dense fibroconnective tissue with no lining cells, consistent with a ganglion cyst (Figure 3).

Postoperatively, the patient was pain-free and showed almost normal strength and sensation in the right leg. On clinical and ultrasonographic examination, no recurrence has been observed up until the time of writing this report.

## Discussion

Ganglia may arise about any joint or tendon, particularly the wrist, the hand and around the ankle joint. These cystic structures lack a synovial lining and may be uniloculated or septated. Histologically, the capsule is composed of collagen fibers and lined by fibrocytes. The content is a gelatinous fluid that is highly viscous secondary to the presence of hyaluronic acid and other mucopolysaccharides [6].

The exact aetiology is unknown; however, traumatic, degenerative or inflammatory processes in adjacent joints have been suggested as possible aetiological factors [7].

When they impinge upon nerves or vessels, ganglia may become symptomatic. However, in the vicinity of the hip, ganglia are associated with unusual and various clinical presentations, and a high index of suspicion is required for predicting the diagnosis.

Akman et al. reported a case of a hip ganglion presenting with symptoms of right sided groin pain [8]. In their case, the diagnosis was established after MRI examination of the right hip region. Inguinal swelling has been reported as another rare presentation of a hip ganglion [9]. Cassina et al. reported a case of a hip ganglion presenting as a pulsatile groin mass [10]. Here, an aneurysm of the femoral artery was first suspected on conventional ultrasound. The true diagnosis of a juxta-articular ganglion was made using colour duplex Doppler ultrasonography and MRI.

Golledge et al. reported a case of a hip ganglion which was initially thought to be inguinal lymphadenopathy, related to a scrotal malignant melanoma excised 2 years earlier [11]. Silver et al. reported three cases of gas-containing ganglia arising from hip joints with advanced degenerative disease [12]. These authors reported this phenomenon as the most specific radiological feature of ganglia around the hip which should be differentiated from overlying bowel gas and other causes of gas within soft tissues.

Bystrom et al. [13] reported a 75-year-old woman with a large palpable mass in the femoral triangle with the symptom of an intermittent, unpleasant sensation of coolness in the right foot. CT and arthrography revealed a large cyst communicating with the hip joint and compressing the femoral vessels ventrally.

Gale et al. reported a case of a hip ganglion with venous compressive symptoms mimicking a deep venous thrombosis [14]. Staneck et al. reported a hip joint ganglion compressing the artery and causing symptoms of peripheral arterial disease and intermittent claudication [15]. Radicular pain is another unusual presentation [16]. Sciatica has been reported secondary to paralabral cysts around the hip joint [17].

Lin et al. reported a hip ganglion which was initially misdiagnosed as a growing inguinal mass (tumour) in a 35-month-old child [18]. In that case, the ganglion was secondary to a labral tear after treatment for left dislocated developmental hip dysplasia.

In our case, as MRI revealed a cystic lesion anterior to the hip joint, we considered a synovial cyst, vascular aneurysm and iliopsoas bursa in the differential diagnosis. Synovial cysts are usually associated with inflammatory arthritis, osteoarthritis, infectious arthritis and post-traumatic degenerative disease [19]. In our patient, the hip joint was normal, and there was no history of previous trauma, making the diagnosis of a synovial cyst less likely. A colour duplex Doppler ultrasonography excluded the possibility of an aneurismal lesion. In such cases, CT-controlled puncture may help establish the diagnosis when a gelatinous substance as the content of the cyst is aspirated.

Ultrasound and colour Doppler ultrasonography are of great importance for diagnosing palpable and non-palpable cystic and soft tissue masses [10]; however, in our patient, the ultrasound was used only to establish the non-vascular nature of the cyst with the diagnosis first established using MRI.

Treatment of ganglion cysts depends on their size and location. Once a ganglion causes compressive symptoms, the treatment is surgical excision [20]. High recurrence rates of 40% have been reported in the literature [21] which has been attributed to inappropriate excision [22]. In our case, no recurrence was observed.

## Conclusion

A high index of suspicion is required to predict non-palpable cystic lesions around the hip joint as they may mimic different disorders and should be kept in mind in the differential diagnosis of unusual groin pain, radicular pain and peripheral vascular disorders.

## Abbreviations

CT: computer tomography; MRI: magnetic resonance imaging; EMG: Electromyography.

## Competing interests

The authors declare that they have no competing interests.

## Authors' contributions

AK and YD performed the operation. AK, YD and ANY were responsible for the pre- and postoperative management. AK, YD and TTS analyzed the literature and wrote the manuscript. ANY edited the manuscript.

## Consent

Written informed consent was obtained from the patient for publication of this case report and any accompanying images. A copy of the written consent is available for review by the Editor-in-Chief of this journal.
